# Genetic diversity and microevolution in clinical *Cryptococcus* isolates from Cameroon

**DOI:** 10.1093/mmy/myad116

**Published:** 2023-11-10

**Authors:** Poppy Sephton-Clark, Elvis Temfack, Jennifer L Tenor, Dena L Toffaletti, Angela Loyse, Síle F Molloy, John R Perfect, Tihana Bicanic, Thomas S Harrison, Olivier Lortholary, Charles Kouanfack, Christina A Cuomo

**Affiliations:** Infectious Disease and Microbiome Program, Broad Institute of MIT and Harvard, Cambridge, Massachusetts, USA; Internal Medicine Unit, Douala General Hospital, Douala, Cameroon; Institut Pasteur, Molecular Mycology Unit, CNRS UMR 2000, Paris, France; Division of Infectious Diseases, Department of Medicine, Duke University School of Medicine, Durham, North Carolina, USA; Division of Infectious Diseases, Department of Medicine, Duke University School of Medicine, Durham, North Carolina, USA; Institute of Infection and Immunity, St George’s University of London, London, UK; Clinical Academic Group in Infection, St George’s University Hospital, London, UK; Institute of Infection and Immunity, St George’s University of London, London, UK; Division of Infectious Diseases, Department of Medicine, Duke University School of Medicine, Durham, North Carolina, USA; Institute of Infection and Immunity, St George’s University of London, London, UK; Clinical Academic Group in Infection, St George’s University Hospital, London, UK; Institute of Infection and Immunity, St George’s University of London, London, UK; MRC Centre for Medical Mycology, University of Exeter, Exeter, UK; Department of Infectious Diseases and Tropical Medicine, Paris Cité University, Necker-Enfants Malades Hospital, AP-HP, IHU Imagine, Paris, France; Mycology Department and National Reference Center for Invasive Mycoses and Antifungals, Institut Pasteur, Paris, France; Department of Public Health, Faculty of Medicine and Pharmaceutical Sciences, University of Dschang, Dschang, Cameroon; Day Hospital, Hospital Central Yaoundé, Yaoundé, Cameroon; Research Center for Emerging and Re-emerging Diseases, Cameroon Baptist Convention Health Services (CBCHS), Yaoundé, Cameroon; Infectious Disease and Microbiome Program, Broad Institute of MIT and Harvard, Cambridge, Massachusetts, USA

**Keywords:** Cryptococcus, genome sequencing, intrahost diversity, GWAS, phylogeography

## Abstract

Cryptococcal meningitis is the second most common cause of death in people living with HIV/AIDS, yet we have a limited understanding of how cryptococcal isolates change over the course of infection. Cryptococcal infections are environmentally acquired, and the genetic diversity of these infecting isolates can also be geographically linked. Here, we employ whole genome sequences for 372 clinical *Cryptococcus* isolates from 341 patients with HIV-associated cryptococcal meningitis obtained via a large clinical trial, across both Malawi and Cameroon, to enable population genetic comparisons of isolates between countries. We see that isolates from Cameroon are highly clonal, when compared to those from Malawi, with differential rates of disruptive variants in genes with roles in DNA binding and energy use. For a subset of patients (22) from Cameroon, we leverage longitudinal sampling, with samples taken at days 7 and 14 post-enrollment, to interrogate the genetic changes that arise over the course of infection, and the genetic diversity of isolates within patients. We see disruptive variants arising over the course of infection in several genes, including the phagocytosis-regulating transcription factor GAT204. In addition, in 13% of patients sampled longitudinally, we see evidence for mixed infections. This approach identifies geographically linked genetic variation, signatures of microevolution, and evidence for mixed infections across a clinical cohort of patients affected by cryptococcal meningitis in Central Africa.

## Introduction

Cryptococcal infections represent a major threat to global health. These infections account for 15%–19% of deaths in those living with HIV/AIDS; the majority of fatal cases of cryptococcal meningitis (between 63% and 75% per year) occur in those living in sub-Saharan Africa.^1^ The predominant species responsible for disease is *Cryptococcus neoformans*.^2^*Cryptococcus neoformans* isolates representing all three major lineages, VNI, VNB, and the rarer VNII, have been isolated from countries across sub-Saharan Africa.^3,4^ While VNI and VNII isolates are found globally, commonly sampled from clinical settings, and found environmentally in pigeon guano, VNB isolates are primarily detected in sub-Saharan Africa from soil and tree sources^4–6^ and only rarely isolated in other regions.^7,8^ Environmental VNB isolates from Botswana display a strong clade-like structure, with two non-recombining lineages encompassing VNB isolates, VNBI and VNBII, defined through phylogenetic analysis.^6^ While VNB isolates are also capable of causing disease, the majority of clinical *C. neoformans* isolates obtained from Uganda, Malawi, Botswana, Zambia, Laos, Thailand, and Vietnam, across multiple clinical trials, belong to the VNI lineage.^4,6,9–11^ Sublineages termed VNIa, VNIb, and VNIc describe the population structure observed for this lineage.^6^

Cryptococcal infections are environmentally acquired, most likely through the inhalation of *Cryptococcus* yeast cells or spores.^12,13^ Cryptococcosis can also arise through the reactivation of a latent infection.^14–16^ This in turn raises the question of whether cryptococcal infections are the result of single or multiple genetically distinct isolates. Longitudinal sampling has shown that the majority (89%–100%) of recurrent infections are caused by a single isolate, based on high genome-wide identity between the incident and recurrent samples.^17,18^ However, studies that analyzed sequence data for multiple colonies from patients at a single time point have shown that mixed infections may be common and are observed in up to 18%–30% of patients when multiple colonies are screened.^19,20^

To assess lineage, population structure, and within-patient relatedness, we sequenced 86 clinical isolates obtained from patients enrolled at the Cameroon site for the multicountry phase III non-inferiority trial, Advancing Cryptococcal Meningitis Treatment in Africa (ACTA).^21^ Based on longitudinal sample analysis, we assess the likelihood of patient infections arising from multiple infecting strains. We compare isolates from Cameroon to those obtained from Malawi in the same clinical trial and across the same timeframe to assess country-linked population structure and identify variants significantly associated with the isolate country of origin.

## Materials and methods

### Sample preparation and sequencing

Cryptococcal isolates (86) were obtained from 57 participants enrolled in the ACTA trial in Cameroon between 2013 and 2016.^21^ A total of 22 participants underwent longitudinal sampling, with samples collected at days 7 and 14, in addition to the baseline day 1 sample. For each timepoint per patient, a single colony was selected for follow-up sequencing. Collected samples were stored at –80°C and grown for two days in 3.5 ml of nutrient-rich yeast peptone dextrose (YPD) (2% yeast extract, 4% peptone, and 4% glucose) media at 30°C and 225 rpm. Genomic DNA was then extracted from a single colony per sample for sequencing with the MasterPure Yeast DNA Purification Kit, as described by Desjardins et al.^6^ DNA was sheared to 250 bp using a Covaris LE instrument and adapted for Illumina sequencing as described by Fisher et al.^22^ Libraries were sequenced on a HiSeq X10, generating 150-bp paired reads.

### Data processing and variant calling

To identify genomic variants, reads were aligned to the *C. neoformans* H99 reference genome (GCA_000149245.3)^23^ with BWA-MEM version 0.7.17.^24^ GATK version 4 variant calling^25^ was carried out as documented in our publicly available cloud-based pipeline (https://github.com/broadinstitute/fungal-wdl/tree/master/gatk4).^26^ Post-calling, variants were filtered on the following parameters: QD < 2.0, QUAL < 30.0, SOR > 3.0, FS > 60.0 (indels > 200), MQ < 40.0, GQ < 50, alternate allele percentage = 0.8, DP < 10. Variants were annotated with SNPeff, version 4.3t.^27^ This annotated variant call file (VCF) was used for genome-wide analysis, with further filtering as described below.

### Population genomic analysis

**Figure 1.**
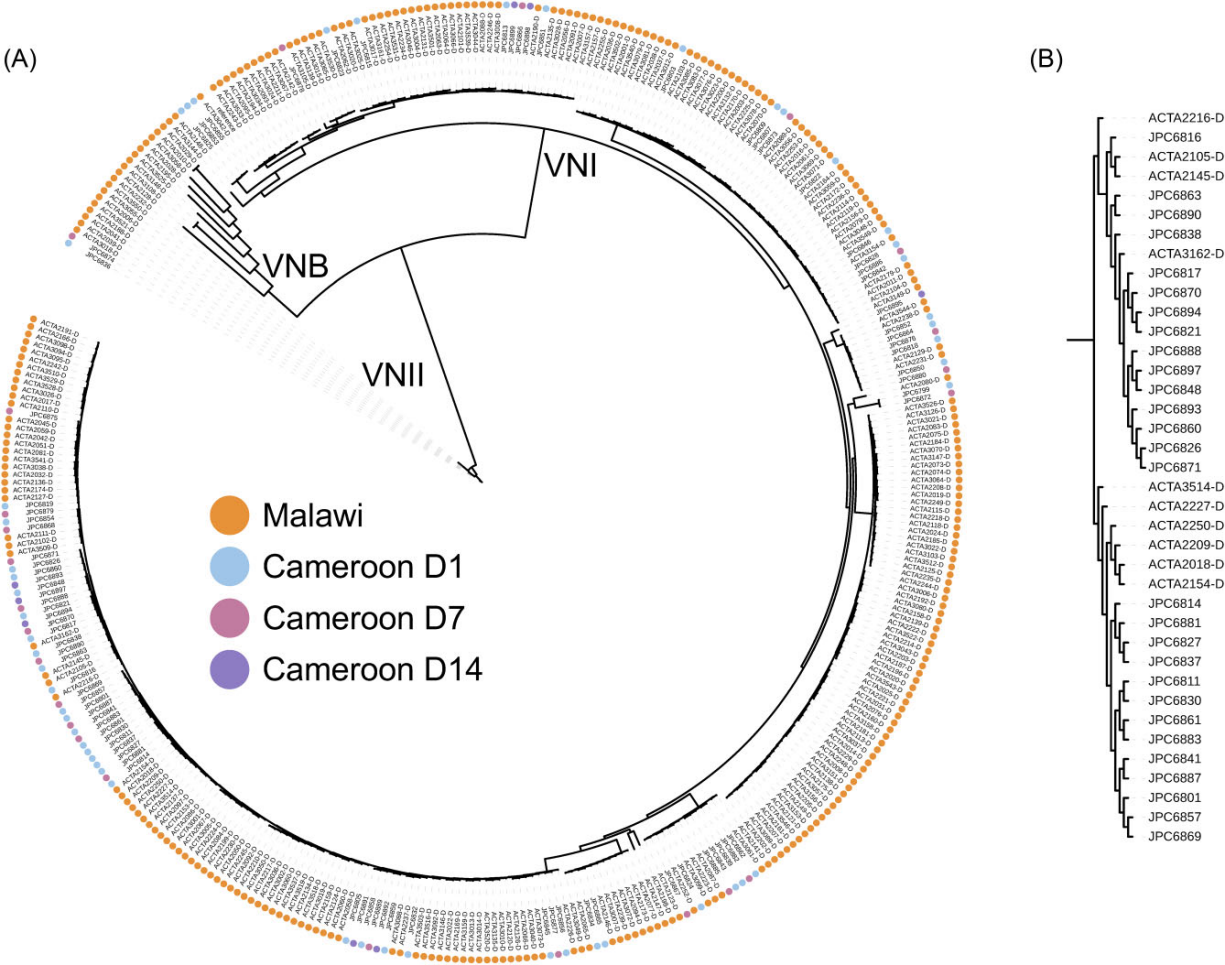
Maximum likelihood phylogeny of isolates from Cameroon and Malawi rooted in the VNII lineage. (A) Phylogeny estimated from segregating SNP sites, with isolates collected on the first day of enrollment from patients in Malawi and Cameroon highlighted in orange and blue, respectively. Isolates collected from patients in Cameroon on days 7 and 14 post-enrollment are highlighted in pink and purple, respectively. (B) Phylogenetic cluster I contains highly related isolates obtained from different patients.

A maximum likelihood phylogeny was estimated using the segregating SNP sites present in one or more isolates (VCF sites), allowing ambiguity in a maximum of 10% of samples, with RAxML version 8.2.12 with GTRCAT rapid bootstrapping,^28^ rooted to VNII isolates as in previous population studies.^6,7^ Isolate lineage was identified based on phylogenetic comparison to isolates previously assigned to lineages from Malawi, which was based on a larger phylogenetic analysis with isolates of known sequence types.^6,11^ In this phylogeny (Fig. 1), the Malawi isolates were grouped into three major clades consistent with their previously assigned lineages (VNI, VNII, and VNB); lineages were assigned to Cameroon isolates based on co-occurrence in these clades. PopGenome (R version 3.5.0, PopGenome version 2.7.5) was used to calculate nucleotide diversity, per chromosome, in 5 kb windows.^29^

### Genome-wide association studies

Association analysis between isolate origins and variants was carried out using PLINK version 1.08p formatted files and Gemma version 0.94.1^30^ (options: centered relatedness matrix gk 1, linear mixed model), as previously described.^6^ Rare variants (present in <5% of the population) were collapsed by gene.

## Results

To assess population structure in clinical isolates obtained from patients in Cameroon enrolled in the ACTA clinical trial, which evaluated the efficacy of two new treatment strategies for cryptococcal meningitis when compared to the international standard, whole genome sequence data for 86 baseline and longitudinal isolates from Cameroon were combined with data for 284 isolates from Malawi, also obtained from patients enrolled in the ACTA trial.^11,21^ Sequences were aligned to the *C. neoformans* (H99) reference genome,^23^ and variants were called and used to infer a maximum likelihood phylogeny (Fig. 1A). Isolates from Cameroon (*N* = 86) were obtained from 57 patients, with 22 of these patients undergoing longitudinal isolate collection at days 7 and 14. Overall, there were 81 *C. neoformans* isolates that belonged to the VNI lineage, 2 that belonged to the VNII lineage, and all *C. neoformans* isolates from Cameroon possessed the MAT alpha locus. Three isolates from Cameroon were excluded from this analysis as they were identified as either AD hybrids (JPC6840 and JPC6884) based on read alignment to all chromosomes of the H99 and JEC21 (GCF000091045.1) genomes or as non-*Cryptococcus* species (JPC6896). Isolates from Cameroon appear highly clonal, with an average terminal branch length across VNI isolates of 0.00070, compared with 0.00085 for VNI isolates (*N* = 266) from Malawi, and a lower average nucleotide diversity across Cameroon VNI isolates of 0.00153, compared to 0.00176 for VNI isolates from Malawi. A single phylogenetic cluster of isolates (Table 1, Fig. 1B) is a major contributing factor to this clonality, as these isolates obtained from different patients appear highly related to one another, with fewer than 20 SNP differences between isolate pairs in this cluster. There does not appear to be a temporal spike in the clonality between these isolates, as these highly related samples were collected between 2014 and 2016; however, this might suggest the presence of a single clone within the environment at this time.

**Table 1.** Highly related non-longitudinal isolates. Isolates from the same phylogenetic cluster (cluster I) are bolded.

Sample 1	Sample 2	SNP differences	Lineage
JPC6866	JPC6898	6	VNI
**JPC6888**	**JPC6817**	8	VNI
**JPC6827**	**JPC6881**	9	VNI
**JPC6870**	**JPC6888**	9	VNI
**JPC6888**	**JPC6893**	9	VNI
**JPC6821**	**JPC6888**	10	VNI
**JPC6826**	**JPC6888**	10	VNI
**JPC6860**	**JPC6888**	10	VNI
**JPC6871**	**JPC6888**	11	VNI
**JPC6888**	**JPC6894**	11	VNI
JPC6889	JPC6891	11	VNI
JPC6898	JPC6899	11	VNI
JPC6858	JPC6889	12	VNI
**JPC6826**	**JPC6860**	19	VNI
**JPC6826**	**JPC6871**	19	VNI

While samples in cluster I appear highly related, this cluster includes isolates from both Malawi and Cameroon. We see that the isolates from Cameroon appear well distributed throughout the phylogeny, despite the geographical separation of these two countries, and independent patient population isolate origins. To assess whether there might be distinct genetic markers of isolates from Cameroon or Malawi, we performed a genome-wide association study (GWAS) analysis to assess variants significantly associated with isolates from either country. Significantly associated with isolates from Cameroon is a missense variant in CNAG_00241, an E3 ubiquitin ligase (Gemma score test *p* = 5.70 × 10 – 5). This variant is present in eight day 1 isolates from Cameroon, in addition to three corresponding day 7 and three corresponding day 14 isolates from Cameroon, and only one isolate from Malawi, and may impact the regulation of protein degradation in these isolates. Significantly associated with isolates from Malawi are two missense variants, one in CNAG_03546, a KEGG ortholog-predicted ATP-dependent DNA helicase, that is present in 55 isolates from Malawi and only 2 isolates from Cameroon (Gemma score test *p* = 2.49 × 10 – 12). The other is a missense in CNAG_05511, an ATPase that is present in 17 isolates from Malawi and 4 isolates from Cameroon (Gemma score test *p* = 3.81 × 10 – 6). These variants may impact DNA binding and energy use in these isolates.

In addition to identifying variants associated with isolates from these two countries, we also interrogated variants arising over the course of infection through variant analysis of the longitudinal isolates from Cameroon. We identified three variants arising over the course of infection in two patients that were predicted to impact gene function (Table 2). Of these, a conservative in-frame insertion in CNAG_03106, a predicted phosphatidylethanolamine-binding protein, arose over 7 days in one patient. Isolates from another single patient developed two variants: a missense variant in CNAG_02166, a DNA-directed RNA polymerase II subunit *RPB1*, and a stop gain in CNAG_06762 (GAT204). This suggests that genes with roles in the regulation of gene expression, including the transcription factor Gat204 that has been implicated in the capsule-independent inhibition of phagocytosis through synergism with Blp1,^31^ may be a source of host microevolution.

**Table 2.** Missense and loss-of-function variants arising in longitudinal isolates.

Sample pairs	Sample days	Gene	Gene function	Variant type
JPC6837/JPC6881	1/7	CNAG_05329	IDH3, myo-inositol 2-dehydrogenase. Likely non-function due to position (385/389 AA)	Disruptive inframe deletion
JPC6837/JPC6881	1/7	CNAG_03106	Phosphatidylethanolamine-binding protein	Conservative inframe insertion
JPC6862/JPC6882	1/7	CNAG_02166	DNA-directed RNA polymerase II subunit RPB1	Missense variant
JPC6862/JPC6882	1/7	CNAG_06762	GAT204 transcription factor	Stop gained

To identify potential instances of mixed infection, we calculated SNP differences between isolates obtained longitudinally from the same patient, and identified three sets of longitudinal isolates that appear to be unrelated to their corresponding primary patient isolates (Table 3). For one patient, the day 7 isolate had 35 854 SNP differences when compared to the day 1 isolate. In another patient, the day 7 isolate has 22 613 SNP differences when compared to the day 1 isolate; however, the day 14 isolate appears highly related to the day 1 isolate (0 SNP differences), suggesting the original isolate was maintained but not sampled at day 7, potentially the result of a mixed infection. In patient C, the day 14 isolate has 44 364 SNP differences when compared to the day 1 isolate; however, the day 7 isolate appears highly similar to the day 1 isolate. These SNP differences identified between longitudinally collected isolates indicate that these may be an instances of mixed-strain infections that can be captured through the isolation and sequencing of multiple colonies per patient over the course of an infection.

**Table 3.** Longitudinal isolates with high numbers of within-patient SNP differences.

Sample	Patient	Day comparison	SNP differences
JPC6827	A	1 vs. 14	35 854
JPC6858	B	1 vs. 7	22 613
JPC6878	B	7 vs. 14	22 602
JPC6891	B	1 vs. 14	0
JPC6857	C	1 vs. 14	44 364
JPC6869	C	7 vs. 14	44 712
JPC6898	C	1 vs. 7	0

## Discussion

Phylogeographic substructures of fungal populations have been observed for multiple fungal pathogens.^32,33^ Here, we compared two cohorts of isolates obtained across the same time period from different countries and geographic regions. We see isolates from Central Africa are highly clonal when compared to isolates obtained from southeastern Africa, and this clonality is predominantly driven by a single phylogenetic cluster. Of note, the highly clonal groupings observed consist of isolates from Cameroon and Malawi, suggesting that highly clonal *C. neoformans* isolates of the VNI lineage may be present across multiple countries in Africa.^34^ We also identify multiple variants significantly associated with either country; given the mixed population structure, these variants are more likely to be linked to the country of origin than driven by the population substructure. Finally, we identify coding variants arising in clinically relevant genes over the course of infection and explore the possibility of multistrain infections through the assessment of longitudinally collected patient samples.

While isolates of the VNI lineage are less genetically diverse than those of the VNB lineage, we observed distinct differences in the levels of nucleotide diversity between VNI isolates from Cameroon and Malawi. Based on measures of population nucleotide diversity, VNI isolates from Malawi appear to be more genetically diverse than those from Cameroon. Previous studies have calculated nucleotide diversity in VNI isolates from a diverse set of countries to be 0.002,^6,7^ which is similar to the values we see for VNI isolates obtained from Malawi and, to a lesser extent, those values for isolates from Cameroon. The presence of highly clonal isolates in Cameroon is not unlike the trends observed in other African countries; for example, isolates from Uganda predominantly belong to a single sequence type (ST93).^9,34,35^ While we observe country-linked differences in diversity here, more extensive sampling by region, and from the environment, is needed to further assess how populations of *Cryptococcus* vary by geographic origin.

Missense and loss-of-function variants were identified in samples from patients after 7 days of infection. These variants are predicted to alter the functions of a phosphatidylethanolamine-binding protein, a DNA-directed RNA polymerase, GAT204, and IDH3. GAT204 has been implicated in virulence, and was shown to be involved in the evasion of phagocytosis.^31^ While variants in these specific genes have not been previously reported, variants in genes with similar functions, including inositol transport and DNA binding, and genes in close proximity to those identified here (CNAG_05330), have been shown to arise over the course of infection.^36^ Multiple studies have identified copy number variation and variants arising in virulence-implicated genes over the course of infection or relapse,^17,18,36,37^ suggesting that this might also be a mechanism of adaptation to the host that sampling of longitudinal isolates offers an important and unique insight into.

The study of mixed infections has been somewhat limited by the practice of sequencing a single colony for follow-up study per patient, with the assumption that an infection may generally be caused by an isolate of a single genotype. The specific sequencing of multiple samples per patient due to longitudinal sampling offers the unique opportunity to assess within-patient isolate heterogeneity. Previous studies that have set out to assess the frequency of mixed strain infections have reported mixed infection rates of 18%–30%, with mixed strains representing different species, serotypes, mating types, and genotypes.^19,20^ While we report a lower proportion of likely mixed strain infections (13%), it is anticipated that the sequencing of multiple colonies per patient, per time point, is needed to better capture the heterogeneity of patient infections. Multiple colony sampling per patient also aids in the evaluation of true mixed infections vs. potential sample swaps. The likelihood of sample swaps occurring here was deemed to be low, given that the phylogenetic analysis did not reveal evidence for sample swaps between longitudinal isolates.

Through the comparison of isolates across two countries, with well-controlled temporal and collection methods, we can assess population structure, diversity, and geographically linked genetic components. Further studies using long-read sequencing could target the additional types of sequence variants missed by short-read sequencing approaches; however, the application of such approaches to population studies is still limited by cost considersations. Future studies leveraging data from multicountry clinical trials will allow for a finer-scale look into geographical population structure, as well as assessing whether there are specific genotypes associated with regions that may be associated with diverse patient outcomes. In addition, studies that include the collection and sequencing of multiple isolates per patient will assess the true state of intra-host pathogen heterogeneity. Together, such studies, linked to clinical metadata, will enable a better understanding of genotype diversity and its contribution to patient outcomes.

## Data Availability

Isolate sequence data can be accessed via NCBI with accession number PRJNA1006382 and PRJNA764746.
